# ‘Physically it was fine, I'd eat what normal people do. But it's never like this in my head’: A qualitative diary study of daily experiences of life in recovery from an eating disorder

**DOI:** 10.1002/erv.3018

**Published:** 2023-08-09

**Authors:** Catherine McCombie, Hannah Ouzzane, Ulrike Schmidt, Vanessa Lawrence

**Affiliations:** ^1^ Department of Health Services and Population Research Institute of Psychiatry, Psychology & Neuroscience King's College London London UK; ^2^ Section of Eating Disorders Department of Psychological Medicine Institute of Psychiatry, Psychology & Neuroscience King's College London London UK; ^3^ South London and Maudsley NHS Foundation Trust London UK

**Keywords:** anorexia nervosa, cognitions, experiences, qualitative, recovery

## Abstract

**Objective:**

High eating disorder (ED) relapse rates stress the need for clearer understanding around how recovery is experienced and maintained. Recent research endorses the concept of recovery as a process rather than an endpoint. This study aimed to investigate daily experiences of living in recovery from an ED.

**Method:**

Fourteen participants who self‐identified as recovered from a formally diagnosed ED were recruited online. A qualitative diary app was used for data collection. Participants completed written or audio open‐ended diary entries every other day for 2 weeks describing their experiences, thoughts, and feelings. Diaries were analysed using reflexive thematic analysis.

**Results:**

Four themes were developed. ‘Ever‐present eating disordered thoughts’ highlights how pervasive these thoughts remain for participants. ‘Impact of social discourses’ unpacks the challenges of maintaining recovery while surrounded by unhelpful social discourses about food and body image. ‘Recovery is precarious’ highlights how a combination of stressors can build up to threaten recovery. ‘Finding balance in recovery’ illustrates the many ways participants try to manage their recovery each day.

**Conclusions:**

The findings make it clear that living in recovery from an ED is a complex process that must be navigated daily. Recommendations for treatment and recovery support are discussed.

## Highlights

- Key findings are: (1) Eating disordered thoughts persist throughout recovery, requiring a substantial amount of time and energy to manage theme; (2) societal attitudes towards food and body image negatively impact recovery; and (3) diaries also illustrated that periods of stress, anxiety, or low mood can trigger more difficult periods of managing recovery, and increase vulnerability to relapse.
- Limitations of the study include: (1) online recruitment; (2) requiring a formal eating disorder (ED) diagnosis; and (3) no assessment of ED treatment after diagnosis, so the impact of treatment received on recovery experiences is not explored.
- Qualitative diary methods may capture unique personality and coping styles in the context of everyday lives, as well as aspects of recovery that are similar across the spectrum of experiences of many people.

## 1 INTRODUCTION

Recovery in eating disorders (EDs) is a contentious subject. There is no agreed definition of, or criteria for, recovery (McDonald et al., 2021), and recovery rates are low: 31.4% for anorexia nervosa (AN) and 68.2% for Bulimia Nervosa at 9 year follow‐up, raising to 62.8% after 22 years for AN (Eddy et al., 2017). Biomedical models of recovery, based on the absence of ED symptoms, dominate the literature and clinical practice (Bardone‐Cone et al., 2018; Elwyn, 2023), despite the existence of recovery models based on lived experience perspectives of recovery that highlight wider criteria and recovery as a journey rather than an end point (Dawson et al., 2014), which tend to be preferred by those who have experienced EDs (Bohrer et al., 2020). ED relapse rates are high, around 40%–50% for AN and 30% for Bulimia Nervosa (Sala et al., 2023), highlighting the challenge for many of maintaining recovery post‐treatment. The effect of currently available treatments on EDs is unclear, with no definitively successful therapies available (Bulik, 2021). A need for treatment focusing on psychological aspects of EDs has been much reported (Murray et al., 2019). This highlights the need for understanding life after treatment and how the psychological aspects of an ED might linger and impact on survivors' wellbeing and susceptibility to relapse.

Findings from interview studies illustrate that recovery is a highly individual, long‐term, and complex process that cannot be captured by outcome measures alone (Stockford et al., 2019). However, there is limited understanding of the day‐to‐day realities of living in recovery from an ED, and how this may contribute to relapse rates and longer‐term impacts of EDs. Hower et al. (2022) highlight a need for using different research methods to more fully understand recovery, including the need to look at recovery over time.

Qualitative research into recovery typical involves interviews or focus groups, which potentially miss nuances of the daily processes that people use to maintain their recovery. Qualitative diary methods could support improving understanding of this, capturing participants' insights as they occur, and allowing experiences to be recorded and tracked across time, enabling patterns to be uncovered (Craig et al., 2017). Keeler et al. (2022) found that those with past or present AN had difficulty retrieving memories, which suggests that capturing experiences as they happen may provide more detailed insights into behavioural, thought, and emotion patterns than those recalled later in interviews.

Understanding day‐to‐day life of ED recovery will help clarify the mental load of maintaining recovery, strategies used to maintain recovery, and potential critical conjunctures that could lead to relapse, with the ultimate aim of informing treatment development and reducing the burden of living in recovery. The aim of this paper is therefore to investigate daily experiences of living in recovery from an ED using qualitative diaries, and to understand the day‐to‐day realities of how people work to maintain recovery.

## 2 METHODS

This study used a qualitative digital diary design, in which participants kept a diary detailing their experiences related to maintaining recovery from an ED. Qualitative diaries were chosen to facilitate identification and understanding of the daily realities of recovery, with app‐based data collection to support participation by providing an easy‐to‐use platform accessible on participants' own devices. The study was designed following recommendations from McCombie et al. (2023) on conducting and reporting qualitative diary methods mental health research.

This study took a relativist ontological position with a social constructionist perspective. This approach was taken as we wanted to look at the experience of being in recovery from an ED from a personal and social perspective: understanding the things individuals do to try and maintain recovery, as well as the social pressures and conflicts that support or hinder recovery, and how participants construct and report their experiences. Social constructionism provides a perspective that is a step away from the medicalised, individualised approach that has tended to dominate treatment and understanding of EDs, and takes into account that EDs are not experienced in isolation from medical and social discourses around food and bodies.

### 2.1 Recruitment and procedures

Participants were recruited online via social media and university research volunteers newsletters. Advert links took interested parties to the study online information sheet and consent form, followed by demographic questions, a screening questionnaire which included questions about diagnosis, time in recovery, and an ED symptom questionnaire. Inclusion criteria were that participants had to be over 18 years old, have a previous ED diagnosis, self‐identify as recovered from their ED, speak English fluently, and not be experiencing an active ED episode.

Screening questions checked the study inclusion and exclusion criteria were met, and the ED Examination–Questionnaire (EDE‐Q; Fairburn & Beglin, 2008) was used to screen out any participants with clinically significant active ED symptoms. The EDE‐Q is a 28 question self‐report questionnaire of ED symptoms over the past 28 days (Fairburn & Beglin, 2008). Participants were included if their global score was below 2.8, a cut‐off widely used to denote the point at which a person is considered to no longer have an ED of clinical severity (Mond et al., 2008).

Twenty six participants completed screening stages. Four were above the EDE‐Q cut off and were excluded from the study. Of the 22 participants who met the criteria for the study, 14 went on to complete diaries. Participant characteristics can be found in Table 1.

**TABLE 1:** Sociodemographic characteristics of participants in eating disorder recovery.

Participant	Age range (years)	Gender	Ethnicity	Diagnosis	Age of ED onset (years)	Length of time in recovery (years)	Diary data collection range (days)
Millie	35–44	F		AN	13	2	1–9
Felicity	25–34	F	Mixed/Multiple ethnic groups	EDNOS	13	5	1–11
Becky	25–34	F	White British	AN	16	5	1–3
Isabelle	35–44	F	White British	AN	13	4	1–5
Cara	20–24	F	Mixed/Multiple ethnic groups	AN	14	2	1–13
Charlie	20–24	M	Asian/Asian British	AN	14	1	1–13
Jake	20–24	M	Asian/Asian British	AN	18	1.5	1–13
Emily	25–34	F	White British	AN	15	7	1–11
Julie	20–24	F	White British	AN	17	1	1–11
Emma	25–34	F	White British	AN	12	7	1–11
May	20–24	F	Asian/Asian British	BED	13	2	1–11
Phillipa	35–44	F	White British	AN	13	21	1–13
Eve	35–44	F	White British	AN	30	1	1–11
Alice	20–24	F	White British	AN	16	6	1–7

Abbreviations: AN, anorexia nervosa; BED, binge eating disorder; EDNOS, eating disorder not otherwise specified.

**FIGURE 1:**
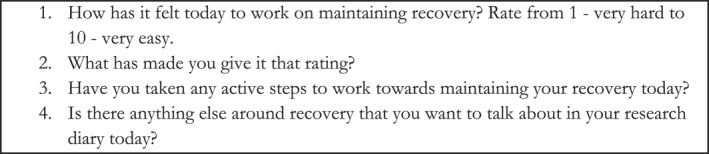
Expiwell daily diary prompts on experiences, thoughts, and feelings related to eating disorder recovery. Expiwell is a personal device app‐based daily diary programme used to collect qualitative data on experiences, thoughts, and feelings related to eating disorder recovery.

Data collection took place via an experience sampling app, Expiwell (Expiwell, 2023). Participants downloaded the app to their smartphones and submitted diary entries every other day for 2 weeks. This pattern and time period was chosen because we wanted to track participants' experiences across a number of days, but daily entries for 2 weeks felt too high a burden. Participants were given a guidance booklet, outlining the research purpose and encouraging them to report whatever they felt was important to them and relevant to their own experiences. The app notified participants when they needed to complete a diary entry, and participants could either audio record or type their entries; most participants used a combination of both. Each diary entry contained the same prompts, which can be found in Figure 1, each presented on a separate screen with space to respond.

All participants provided informed consent before taking part in the study. Consideration was given to the potential for distress arising from keeping diary entries about ED recovery, so the diary keeping guidance included suggestions no more than 5–10 min is spent on each entry, and a reminder that the diary should not be used as a food or exercise journal or place for seeking help. Guidelines included details of how to access support, when it might be helpful to reach out for support, and advice to contact the researchers if they felt that taking part was having a negative impact on their mental health. In awareness of the burden of taking part in a diary study, the guidance also included reassurance that it was fine to miss some diary entries. Participants were able to review and edit their diary entries before submitting them, to give them control over what was submitted in the event they felt that after recording something they did not want to share it. The study was approved by the King's College London Health Faculties Research Ethics Subcommittee, reference HR/DP‐21/22‐26071.

Data were analysed in NVivo 12 (QSR International, 2018), using Reflexive Thematic Analysis (Braun & Clarke, 2022) with some narrative elements incorporated (Andrews et al., 2008) to capture the changes seen over time in participants' diaries. Two researchers (CM and HO) independently familiarised themselves with the data and conducted initial rounds of coding. Meetings then took place to discuss and review the data and coding with a third researcher (VL) to begin to identify and formulate themes. Discussions around data, coding, theme formulation, and the final themes, were focused on ensuring that multiple perspectives were captured in the analysis and that the final themes reflected the key concepts within the data and the nuances associated with each.

During analysis, we were interested in how each participant constructed their experience of recovery, and in the language and discourses that were used in these constructions. We aimed to ensure that participants words and language were the centre of the analysis, rather than preconceptions from prior research. Data were analysed line‐by‐line with an initial focus on capturing the general experiences and tone of each participants' diary. Subsequent read‐throughs and coding sessions focused on concepts around recovery and EDs, and language in how participants relate these to themselves and their experiences. Narrative analysis elements were included at this stage, to track concepts across individual participants' diaries and see how events and experiences played out across time, before considering themes across participants. Themes were developed iteratively throughout analysis, with some developed further and others put aside following further analysis and discussion.

CM and HO conducted the main analysis, and reflected on and acknowledged their own perspectives throughout the research and analysis process. CM is a researcher with a background in mental health practice in the NHS, and has her own experience of recovery from an ED. HO is a neuroscience student specialising in neuropsychology, and has an understanding of generalised anxiety disorder through personal experience and support groups. Researchers kept reflexive journals throughout the study and analysis, and held several meetings to discuss the process, and to support researcher reflexivity in discussing alternate perspectives.

## 3 RESULTS

Four themes were developed, each capturing an element of life in recovery from an ED. To provide context for quotes, participant characteristics are outlined in Table 1, along with the day ranges each participant submitted diary entries.

**FIGURE 2:**
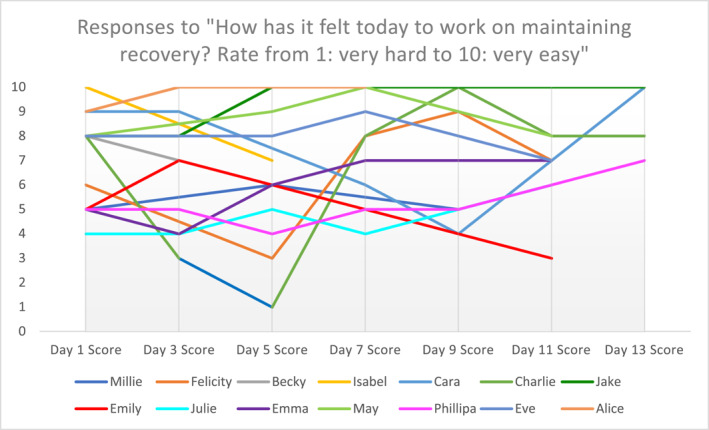
Participant experiences of managing their recovery over study follow up. Responses to the question “how has it felt today to work on maintaining recovery? Rate from 1‐very hard to 10‐very easy”. Diary score data collection ranges: Millie (day 1–9); Felicity (day 1–11); Becky (day 1–3); Isabelle (day 1–5); Cara (day 1–13); Charlie (day 1–13); Jake (day 1–13); Emily (day 1–11); Julie (day 1–11); Emma (day 1–11); May (day 1–11); Phillipa (day 1–13); Eve (day 1–11); Alice (day 1–7).

Participants reported a wide range of experiences of managing their recovery. Responses to the question, ‘How has it felt today to work on maintaining recovery? Rate from 1–very hard to 10–very easy’, are shown in Figure 2, illustrating how these changed across the study period.

### 3.1 Theme 1: Ever‐present ED thoughts

This theme highlights the continuous presence of ED thoughts in participants' lives, and the measures they take to try to manage these. Participants reflected that even when they were managing to eat healthily, thoughts of food restriction, exercising, guilt and shame around food, and anxiety about eating remain.

I’d eat, I guess, what normal people do. But… it’s never like this in my head. I always have thoughts of like… skipping some meals, or maybe I shouldn’t… or…. Yeah. I would like not to have them.

(Emma).

One participant's language hints at the effort involved in managing these thoughts and limiting the impact of them on their behaviour–‘*I managed to avoid overexercising and I didn't skip meals*’, (Becky). The repeated mention of ED related thoughts across diary entries and participants emphasises that they are still very prevalent even in recovery, and take up a lot of participants' headspace and energy. References to ongoing guilt around eating were seen across all participants, sometimes evidenced by the noted absence of these thoughts–‘*Basically no guilt today*’, (Julie).

Most participants talked about having to justify eating to themselves in some way, often framed as acute awareness of a need to balance food intake against exercise–‘*I skipped a meal… [so] I tried to limit my physical activity…*’ (Becky). Several participants talked about food in a way that illustrated that they eat in order to be able to do things–‘*I made sure I had enough fuel to go skating. I distracted myself from negative thoughts by doing things I know I enjoy.*’ (Cara). Despite this, higher calorie foods were associated with guilt and shame, which then affected how participants felt for the rest of the day, and sometimes days later (as evidenced in subsequent diary entries)–‘*I feel guilty for having something sweet… I had difficulty deciding whether I should have meals but ultimately decided to have them*’ (Charlie).

Alongside these thoughts, many participants also reported heightened awareness of their bodies and themselves in space, beyond passing self‐consciousness:

“I felt really, really self‐conscious walking into the room, there was a lot of food out, they did like a big buffet thing for us and I felt really, really big all of a sudden um and I felt like that today as well,”

(Millie).

Managing the constant presence of ED thoughts required daily effort from participants. Many talked about keeping busy to distract themselves from these thoughts. Some referenced the struggle to manage ED thoughts on days that were quieter–‘*I struggle with nothing days… When I'm busy it's easy to be distracted and feel deserving to eat, but it's exhausting.*’ (Cara). This participant also mentioned that keeping busy can be a double‐edged sword, as then thoughts of being too busy to eat can creep in, which then need tackling. Another participant mentioned overworking in order to distract herself. Busyness as a key coping strategy for managing eating disordered thoughts could leave people vulnerable to relapse in less busy periods when they struggle more to justify eating. The many references to keeping busy to distract from thoughts suggests that participants felt under‐equipped to manage these thoughts in other ways.

Several participants felt the inevitability of the long term presence of ED thoughts ‐ “*I don't think the thoughts may go away necessarily where I don't think about restricting or binging ever again.*” (Charlie) and that “*even if the physical and behavioural elements are moved on from, the remnants of that remains. I think that’s when we need to keep being kind to ourselves and each other and those parts of the e.d are what we need to have more conversations about*.” (Felicity). These thoughts seemed reflective of all participants' experiences, in terms of the presence of ED thoughts in their daily lives.

### 3.2 Theme 2: Impact of social discourses

This theme highlights the way social aspects of participants' lives, and social discourses around eating and bodies, affect participants' recovery journeys. Social interactions were a source of support and positivity for some. However, interactions and cultural discourses around food and bodies were a frequently a source of stress, and challenged participants' attempts to distance themselves from eating disordered thoughts.

Being around other people eating was often reported as a source of stress and anxiety across participants and diary entries. This could arise from portion size comparisons, which were mentioned by several participants and had a negative impact on engagement with and enjoyment of social occasions, ‘*I can sometimes compare my portions to everyone in my life. It can be hard to focus.*’ (Charlie). One participant, starting a new job, writes:

"I am finding it quite difficult because everybody eats which I didn’t kinda anticipate for. Everybody is eating in the same area, in the same small kitchen, sharing food… I just found that really difficult… it is still making me quite anxious.”

(Millie).

Alongside the usual anxieties of starting a new job, anxiety around social eating and expectations seems to be exacerbated. This adds another factor to social events that make these harder to manage in recovery from an ED, and means that those in recovery may avoid or struggle through gatherings involving food. For several participants, being around others also made it harder to focus on their own needs, as reflected in one participant's comment–‘*I find time alone with myself therapeutic because I'm not influenced by other factors, and also can hear what my needs are*’ (Alice). One participant mentions trying to cope with social events by ‘*Eating what my body needs and not looking at other people's plates*’ (Eve), indicating the need to manage anxiety and unhelpful thoughts around social eating.

General social dialogue around food and bodies was particularly challenging for one participant:

“Girls especially talk a lot about body image and ask about their own bodies like ask for your opinion, and that can be a bit challenging because it causes you to reflect more on your own body… one of them said that she is going to start going to the gym because she wants [to] look skinny and change the way she looks and that can be quite challenging because it kind of reinforces the idea that that’s what I should be doing… it is quite difficult because girls or people generally aren’t happy with their bodies, and body acceptance isn’t a massive thing, so trying to achieve that is really challenging.”

(Cara).

This highlights the difficulty of trying to maintain recovery and positive body image in a world where one type of body is still idolised by a majority, and where being openly self‐critical about one's appearance is normalised.

Social events involving food were often anticipated with anxiety–‘*I'm a bit anxious about the Christmas period coming up and weight gain etc. but also reminding myself that it’s not the be all and end all*’ (Isabel). Following social events, alongside heightened anxiety from the event itself participants reported struggling more with challenging eating disordered thoughts–‘*hard after a hen do of eating/drinking more than usual to not give into the urge of restriction*’ (Emily). This suggests that participants' anxiety beforehand is not just from the situation itself, but from awareness that the time afterwards will be more difficult in terms of managing ED thoughts, which is likely to make individuals anxious about and reluctant to engage in social events.

Several participants also referenced the support that social interactions can provide. ‘*I have a tendency to revert to bad habits when stressed, and a lack of thorough support system*’ (Julie). This suggests that social support can help participants manage ED symptoms, and that, in combination with other stressors, a lack of social support can further exacerbate challenges to recovery. For other participants, eating dinner with friends was ‘*an easy way for me not think too much regarding my appetite or weight. I have better moods so can eat better*’ (May), and a way to enjoy time with family, ‘*Enjoying sweet foods with my daughter*’ (Eve).

### 3.3 Theme 3: Recovery is precarious

The previous themes illustrate that most participants felt the ED was lurking in the background. Consistent with this was a strong sense across diaries that recovery is precarious and that participants have many challenges to recovery that they must contend with in their daily lives. It seems that there were several events or factors that meant the ED slipped from the background to the forefront of participants' minds.

Heightened times of generalised anxiety and stress were a flash point for the increased presence of ED thoughts for several participants. They were often acutely aware of how difficult this was making recovery: ‘*The thoughts about restricting are really, really intense at the moment, but I think that is to do with the anxiety around everything and lots of changes happening*’, (Millie). Alongside increased anxiety leading to more ED thoughts, having stressful events to cope with meant less energy was available for the work of recovery–‘*I have had university exams recently which have definitely made a few meal and snack times a bit more difficult. I have another exam tomorrow which I'm not looking forward to, my anxiety really shoots up around them! It definitely can put me off challenging myself more in my ED recovery*’ (Phillipa).

For some participants, periods of low mood made recovery more challenging due to lack of motivation to manage recovery or reduced appetite. For others, low mood had a different effect–‘*I find it difficult when I feel at my most numb to not turn to food restriction or food binges not to make me feel some sense of relief or self‐accomplishment given how low I can feel due to mental health*’. (Charlie). This participant also references a battle around how lack of appetite from depressive episodes is welcomed as they then think about food less, but that this in turn brings another challenge as they then have to think more about managing their food intake. It seems that there is not a straightforward relationship between mood and recovery, and complexities around this in itself can make recovery more of a struggle. Another participant referenced how being in a good mood made it easier to manage recovery, and reflected that ‘*I finished some work today and felt positive, it may be one reason I can keep smile/keep eating well… I know having a stable mood helped me get rid of eating disorder*’ (May). These comments make it clear that mood and anxiety have a big impact on managing and maintaining recovery, and that periods of low mood or heightened anxiety can be a flash point for relapse.

We can see from the diaries that a bad day in terms of mood, stressful events, difficult social situations, or general anxiety can lead to several days of recovery being more difficult to maintain. These quotes from one participant across 5 days illustrate this:

It’s finally getting to the point where I don’t think about food/exercise/body image continuously. I’m ambivalent to say I’m completely recovered as I’ve said that before and then had bad relapses later on, but I feel I’m doing well at the moment.

(Cara).

Several days later, following a difficult social event–‘*It's been a tricky day. I've felt low and very sensitive. Didn't feel motivated or deserving… I've been struggling with body image recently. I keep comparing my body to my friends'. I feel like I'm not getting enough exercise my gym has been closed*’.

This theme suggests that recovery is a state of fine balance that can be brought out of kilter relatively easily by difficult events, general stresses, and running out of the energy needed to keep a constant monitor on thoughts and feelings to do with eating and the ED.

### 3.4 Theme 4: Finding balance in recovery

This theme considers the work that participants put into recovery each day, trying to find balance between treating food as an adversary to be managed, and treating themselves with compassion and as individuals with their own specific needs. Key strategies used by most participants included ‘*speaking kindly to myself*’ (Isabel), ‘*listening to my body's needs*’ (Eve), and having goals to focus on–‘*When I've got things to do I kinda feel more purposeful, like I need food more to be able to concentrate and get through what needs to be done*’. (Cara). Focus on goals was often mentioned as a way to justify eating, and seemed to be a way to take focus away from food and instead focus on recovery. EDs were referenced as something that would get in the way of life goals, and so goals represented a key way in which participants re‐committed to recovery on a regular basis.

One participant mentions ‘[I] *haven't had that many anxieties, but also haven't really TRIED to be better*’ (Emma), which suggests that some days, taking active steps to support recovery might be too difficult, perhaps in terms of motivation. Others talked about the importance of questioning themselves ‘*I've been rethinking my eating disorder and how important it is to question myself and my actions*’. (Felicity), and acknowledging difficulties–‘*I've had to acknowledge that I still have rules surrounding my eating and I still have to think if I should eat even when I'm hungry. At least I win that argument with myself more and more these days*’. (Charlie). Being aware of potential triggers was a recurring topic, which sometimes could be comments from friends or relatives–‘*Some of my biggest triggers are when I'm interacting with my mum. I have to not let her comments or negativity affect me too much that I take it out on myself*’. (Alice).

I experienced some difficulties at lunch, where I felt quite anxious about how much to eat given that I was having a very early dinner today. I felt like maybe I should think about having less. In the end I did have enough today overall, which I feel very positive about.

(Phillipa)

The above quote illustrates the many internal debates and decisions that participants reference regarding how much to eat, and how often they need to re‐commit to recovery. Similarly, a commitment to recovery enabled one participant to manage a social event ‘*Worrying less about what other people think of my food choices and more what I think about them*’ (Eve). It seems that a lot of managing recovery focuses around self‐doubt over decisions related to food, creating internal conflict and leading to increased anxiety and susceptibility to relapse if self‐confidence is otherwise challenged.

## 4 DISCUSSION

The findings illustrate the complex day‐to‐day realities of life in recovery from an ED. In the theme ‘ever‐present eating disordered thoughts’, we see how pervasive these thoughts can be, and the seemingly exhausting ways in which participants try to manage them. ‘Impact of social discourses’ unpacks the challenges of maintaining recovery while surrounded by unhelpful discourses about food and body image, as well as the ways that social support can help reduce the impact of these. ‘Recovery is precarious’ highlights how a combination of factors and stressors can build up to threats to recovery, and how these then need to be actively managed at a time when participants resources may be lower. Finally, ‘Finding balance in recovery’ focuses on the coping strategies that participants use to try and find balance between self‐compassion and acceptance, and treating food and their bodies as an adversary. In whole, the findings make it clear that living in recovery from an ED does not represent freedom from the ED but remains a complex process that must be navigated daily.

The continued presence of eating disordered thoughts in participants' lives, and the amount of energy and time put into managing these in the form of distraction and keeping busy, has significant implications for quality of life and treatment development. Recovery research highlights a need to find a new identity beyond the ED (Kenny & Lewis, 2021), but with so much time and energy focused on battling recurring ED thoughts, or simply trying to distract from them, the energy left for reflection and reconstructing identity free from the ED must be limited. The ‘impact of social discourses’ theme, alongside participants struggle to balance food intake and exercises, supports work by LaMarre and Rice (2016) stating that normal eating is counter‐culture, and that recovery ‘instructions’ gained through therapy focus on ways to be healthy that directly oppose dominant health discourses. These discourses represent an additional layer of difficulty in achieving lasting and meaningful recovery.

The current findings shed further light on research that indicates lasting negative social and psychiatric impacts of EDs (Dobrescu et al., 2020), as they demonstrate in recovery, there are still huge daily impacts on wellbeing and social lives. In their work on mental health recovery narratives, Llewellyn‐Beardsley et al. (2019) identified that there are factors that help and hinder recovery at individual, socio‐cultural, and systemic levels, and this is seen in the current findings. Research into the EDs Recovery Endorsement Questionnaire found four key components of recovery: lack of symptomatic behaviour, acceptance of self and body, social and emotional connection, and physical health, that highlight the importance of the psychosocial and emotional aspects of recovery (Bachner‐Melman et al., 2018). The current findings suggest that participants felt under‐equipped to achieve lasting recovery in all four areas.

This study therefore has important implications for treatment. It is clear that support is needed in managing stress, anxiety, and low mood, and that these are all essential to treatment. Participants made it clear that they felt they had been left to manage thoughts by themselves, and that ED thoughts had the biggest impact on their lives in recovery. Alongside this, the prevalence of unhealthy attitudes towards food and bodies, and the struggle to avoid engagement with these, suggests that mindfulness based recovery programmes may be promising. McDonald et al. (2021) found that both ED survivors and EDs therapists feel that recovery is a journey rather than an endpoint, but while recovery is always in process, it seems from the current findings that there are limits to the energy and time that people in recovery can put into other things. If recovery is to be lasting and meaningful, treatment needs to move the focus from managing food towards supporting people to manage mood, emotions and thoughts.

There are some limitations in the study. Firstly, online recruitment excludes a potential group of participants who are online less and may have different characteristics and experiences. Requiring a diagnosis (although unverified by the research team) also excludes people who have valid experiences of EDs and consider themselves recovered but did not receive formal diagnosis. Additionally, we did not ascertain whether participants had previously received treatment for their ED after diagnosis; although several mentioned past treatment in their diary entries. Nonetheless, in asking participants to self‐identify as recovered we were able to capture experiences of participants across a range of recovery stages and experiences, and find aspects of recovery that were similar across the spectrum of experience.

The qualitative diary app is a key strength, as this enabled detailed recovery experiences across time to be captured. By recording their daily life in this way, we were able to get a strong sense of what participants' day‐to‐day reality was like, as well as each participants' unique personality and coping styles, in the context of their everyday lives. Considering the need for greater understanding of both active EDs and recovery, qualitative diary methods are well placed to make substantial contributions to the ED research base by putting participant experiences at the absolute centre of the research.

## CONFLICT OF INTEREST STATEMENT

There are no conflicts of interest to declare.

## Supporting information

TOC Entry

## Data Availability

Data is qualitative and we do not have ethical permission to share.
