# Supplementary material for: ‘Physically it was fine, I'd eat what normal people do. But it's never like this in my head’: A qualitative diary study of daily experiences of life in recovery from an eating disorder
Source: Eur Eat Disord Rev. 2023 Aug 9;32(1):46–55. doi: 10.1002/erv.3018 (PMC10952333; doi:10.1002/erv.3018)

**“Physically it was fine, I’d eat what normal people do. But it’s never like this in my head”: A qualitative diary study of daily experiences of life in recovery from an eating disorder**

Catherine McCombie*, Hannah Ouzzane, Ulrike Schmidt, Vanessa Lawrence

Thematic analysis findings characterise the day-to-day challenges of maintaining recovery. “Ever-present eating disordered thoughts” highlights how pervasive these thoughts remain for participants, while “Impact of social discourses” unpacks the challenges of maintaining recovery while surrounded by unhelpful social discourses about food and body image. “Recovery is precarious” highlights how a combination of stressors can build up to threaten recovery, and “Finding balance in recovery” illustrates the many ways participants try to manage their recovery each day.


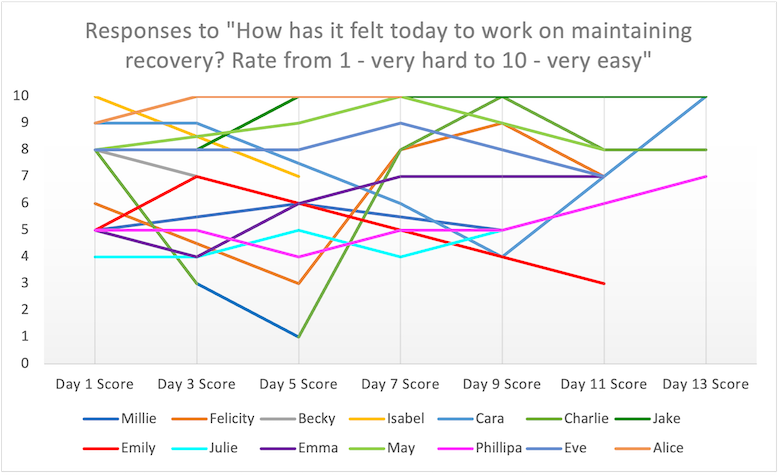

Supplement: Supplementary file 1 — TOC Entry [file ERV-32-46-s001.docx]
